# Combined analysis of the non-pneumatic anti-shock garment on mortality from hypovolemic shock secondary to obstetric hemorrhage

**DOI:** 10.1186/1471-2393-13-208

**Published:** 2013-11-15

**Authors:** Alison M El Ayadi, Elizabeth Butrick, Jillian Geissler, Suellen Miller

**Affiliations:** 1 Bixby Center for Global Reproductive Health, Department of Obstetrics, Gynecology & Reproductive Sciences, University of California San Francisco, 50 Beale Street, Suite 1200, San Francisco, CA 94105, USA

**Keywords:** Obstetric hemorrhage, Hypovolemic shock, Non-pneumatic anti-shock garment, NASG

## Abstract

**Background:**

Obstetric hemorrhage is the leading cause of maternal mortality, particularly in low-resource settings where women face significant delays in accessing definitive treatment. The Non-pneumatic Anti-Shock Garment (NASG) is a first-aid device to stabilize women in hypovolemic shock secondary to obstetric hemorrhage. Prior studies on the effectiveness of the NASG have suffered from small sample sizes and insufficient statistical power. We sought to generate a summary effect estimate of this intervention by combining data from all previous quasi-experimental studies.

**Methods:**

Five quasi-experimental studies that tested the NASG as treatment for hypovolemic shock secondary to obstetric hemorrhage at the tertiary care facility level were included in the analysis. We evaluated heterogeneity of effect across studies and calculated pooled odds ratios. We also conducted a subgroup analysis among women in the most severe condition.

**Results:**

Participant characteristics were similar across studies with some variation in hemorrhage etiology. Median blood loss was at least 50% lower in the intervention group than the control group. The pooled odds ratio suggested that NASG intervention was associated with a 38% significantly reduced odds of mortality among the overall sample, and a 59% significantly reduced odds of mortality among the most severe women.

**Conclusions:**

The results from this combined analysis suggest that NASG intervention is associated with a reduced odds of death for women with hypovolemic shock secondary to obstetric hemorrhage. Further research should focus on application of the NASG at the community or primary health care level, and utilize a more robust methodology.

## Background

Obstetric hemorrhage (OH), including postpartum hemorrhage (PPH), is the leading cause of maternal mortality worldwide, particularly in low-resource settings where access to blood and surgery are limited. About 30% of direct maternal deaths are caused by OH, the vast majority of which occur in developing countries [1-3]. While administration of prophylactic uterotonics reduces the risk of atonic PPH by up to 60% [4-6], thousands of women still experience PPH, and die without rapid recognition and treatment. Further, not all uterine atony will respond to uterotonic treatment of PPH, and not all OH etiologies will respond to uterotonics.

**Figure 1:**
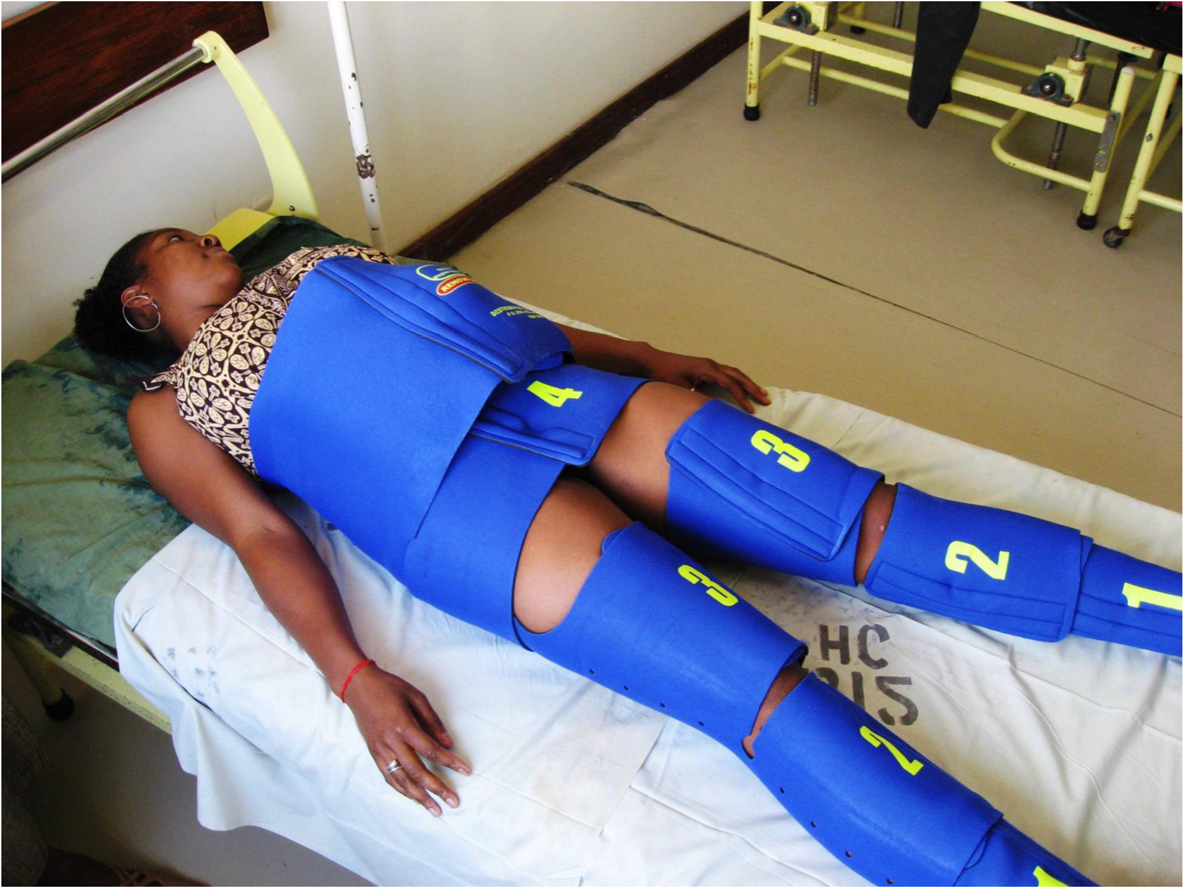
Non-pneumatic Anti-Shock Garment.

In low-resource settings, a series of delays contribute to high maternal mortality: the decision to seek care, procuring transport and reaching a Comprehensive Emergency Obstetric Care (CEmOC) facility, and obtaining quality definitive care [7]. One new low-technology first-aid device for stabilizing women suffering hypovolemic shock secondary to obstetric hemorrhage is the Non-Pneumatic Anti-Shock Garment (NASG), a lower-body compression garment made of neoprene and Velcro™ (Zoex Corporation, Colma CA, USA; Figure 1). The NASG plays a unique role in hemorrhage and shock management by reversing shock and decreasing blood loss thereby stabilizing the woman until definitive care is accessed.

Previous studies of the NASG at tertiary care facilities have shown significantly reduced measured blood loss, more rapid recovery from shock, and decreased mortality [8-12]. However, because mortality from obstetric hemorrhage is rare, these studies have small sample sizes and lack statistical power for some outcomes. These same characteristics make conducting further studies difficult and expensive. Therefore, we sought to combine all the available data on NASG effectiveness, all of which was conducted by our research group or our collaborators, utilizing meta-analytic procedures to provide a summary effect estimate.

## Methods

Research on the NASG has been spearheaded by our research group, the Safe Motherhood Program at the University of California, San Francisco (UCSF). Our research program on the NASG is called Lifewrap (http://www.lifewrap.org), and is a 501c3 educational organization. All of the authors are employees of UCSF, and none has a financial interest in the NASG product. We confirmed that no other studies of the NASG existed in the literature by searching electronic databases and relevant conference proceedings utilizing the terms “non-pneumatic anti-shock garment”, “anti-shock garment” and acronym, “NASG” through August 31, 2012, and by conferring with researchers and non-governmental organizations working with the NASG. Our analysis comprised five quasi-experimental (non-randomized) studies of the NASG as treatment for hypovolemic shock secondary to obstetric hemorrhage conducted by our research group at UCSF or in conjunction with collaborators: Miller 2006 in Egypt [8], Miller 2010 in Egypt and Nigeria [12], Magwali 2012 in Lusaka, Zambia and Harare, Zimbabwe [13]; Miller 2012 in Copperbelt, Zambia [14]; and Maknikar 2012 in India [15]. We excluded one study, Jega 2012 in Nigeria, because there was no comparison group [16]. Further articles identified referred to the aforementioned Miller 2006 [10] and Miller 2010 [9,17-21] studies. The methodological quality of the five included studies was assessed. We also had access to the full datasets for four of the five studies. Two of the included trials were conducted within the same parent study as preparatory phases for a cluster-randomized controlled trial of the NASG (Magwali 2012, and Miller 2012) but are presented separately in this review because the Miller 2012 site began 18 months later than Magwali 2012 and had different characteristics.

All trials had a quasi-experimental design, utilizing a pre-intervention phase followed by NASG intervention phase, except for Maknikar 2012, which enrolled similar individuals into control and treatment arms concurrently. Eligibility criteria were similar across trials; women with hypovolemic shock secondary to obstetric hemorrhage from any etiology, an estimated blood loss of ≥750 mL (Miller 2006 and Miller 2010) or ≥1000 mL (Magwali 2012, Miller 2012, and Maknikar 2012) and one or more clinical signs of hypovolemic shock (systolic blood pressure (SBP) ≤100 mmHg and/or pulse ≥100 beats per minute (BPM)). Standard protocols for hemorrhage and shock were followed in both phases: administration of crystalloid intravenous fluids and blood transfusion, use of uterotonics, uterine massage, vaginal procedures, and surgery. Mortality as outcome was reported for all studies. Four studies reported blood loss measured by a closed-end calibrated plastic drape; one study did not capture blood loss (Maknikar 2012).

The odds ratios and 95% confidence intervals were calculated for each study, and meta-analytic procedures were utilized in Stata (v.11, College Station, TX) to synthesize a pooled odds ratio (POR) utilizing the Mantel-Haenszel fixed effects method. This method was chosen *a priori* because it is more robust for smaller studies and low event rates, and the assumptions are theoretically more appropriate for the NASG intervention [22,23]. We tested for heterogeneity of effect using the chi-square test for heterogeneity (Q statistic), using a type 1 error threshold of 10% for statistical significance, and evaluated the I^2^ value and 95% confidence interval to quantify the degree of heterogeneity. We then conducted a subgroup analysis among participants in the most severe condition who entered the study unconscious or with mean arterial pressure (MAP) <60 mmHg [24]. Where heterogeneity of effect was statistically significant, we evaluated a random effects model utilizing the DerSimonian and Laird procedure [25]. We also conducted a sensitivity analysis for the overall sample and subsamples by estimating pooled effect estimates, sequentially excluding each study to assess whether results obtained in the primary analysis were robust to the exclusion of particular studies.

## Results

Characteristics of the five trials are presented in Table 1. Trail size ranged from 260 (Maknikar 2012) to 1442 total participants (Miller 2010) representing 3,563 participants; 1616 received NASG treatment plus standard OH care (45.4%) and 1947 received standard OH care only (54.6%). The mean age of participants was 28.1 (range: 26.3 – 29.3) and mean parity was 2.7 (range 1.8 – 3.3). Four studies reported hemorrhage diagnosis, which varied somewhat across studies, but were largely postpartum etiologies or complications of abortion. Median estimated blood loss at study entry ranged from 700 – 1200 mLs. The proportion with MAP < 60 varied across studies, from 13.5% (Miller 2006) to 44.6% (Magwali 2012).

**Table 1:** Selected characteristics of study participants for studies included in combined analysis of NASG, all study participants n = 3563

	**Miller 2006**		**Miller 2010**		**Magwali 2012**		**Miller 2012**		**Maknikar 2012**^**b**^	
	**Control**	**NASG**	**Control**	**NASG**	**Control**	**NASG**	**Control**	**NASG**	**Control**	**NASG**
	**n = 158**	**n = 206**	**n = 607**	**n = 835**	**n = 469**	**n = 306**	**n = 574**	**n = 148**	**n = 139**	**n = 121**
Demographics										
Age mean (SD)	27.3 (6.1)	27.4 (5.7)	29.0 (6.4)	29.3 (6.2)	28.0 (6.1)	27.9 (6.3)	26.7 (6.9)	26.5 (6.9)		
Parity mean (SD)	2.2 (2.2)	1.8 (1.8)	3.2 (2.8)	3.3 (2.8)	2.2 (1.7)	2.4 (1.9)	2.3 (2.2)	2.2 (2.1)		
Weeks pregnant^a^ mean (SD)	37.9 (2.8)	38.1 (2.9)	37.4 (3.2)	37.6 (3.2)	36.7 (3.8)	37.2 (3.5)	36.3 (2.8)	36.4 (2.7)		
Definitive diagnosis										
Uterine atony	67 (43.0)	69 (33.5)	190 (31.3)	319 (38.2)	92 (19.6)	65 (21.2)	68 (11.8)	21 (14.2)		
Complications of abortion	25 (16.0)	35 (17.0)	45 (7.4)	93 (11.1)	146 (31.1)	89 (29.1)	95 (16.6)	26 (17.6)		
Placenta previa	4 (2.6)	6 (2.9)	40 (6.6)	31 (3.7)	16 (3.4)	12 (3.9)	33 (5.8)	4 (2.7)		
Placental abruption	4 (2.6)	11 (5.3)	79 (13.0)	98 (11.7)	45 (9.6)	30 (9.8)	36 (6.3)	8 (5.4)		
Ectopic pregnancy	9 (5.8)	19 (9.2)	95 (15.7)	85 (10.2)	26 (5.5)	9 (2.9)	20 (3.5)	7 (4.7)		
Molar pregnancy	3 (1.9)	5 (2.4)	7 (1.2)	11 (1.3)	2 (0.4)	2 (0.7)	3 (0.5)	0 (0.0)		
Ruptured uterus	5 (3.2)	7 (3.4)	46 (7.6)	32 (3.8)	30 (6.4)	16 (5.2)	19 (3.3)	5 (3.4)		
Placenta accreta	0 (0.0)	0 (0.0)	6 (1.0)	9 (1.1)	2 (0.4)	2 (0.7)	3 (0.5)	2 (1.4)		
Lacerations/Genital trauma	15 (9.6)	29 (14.1)	25 (4.1)	65 (7.8)	26 (5.5)	14 (4.6)	166 (28.9)	38 (25.7)		
Retained placenta	9 (5.8)	12 (5.8)	71 (11.7)	83 (9.9)	82 (17.5)	64 (20.9)	127 (22.1)	36 (24.3)		
Other	15 (9.6)	13 (6.3)	3 (0.5)	9 (1.1)	2 (0.4)	2 (0.7)	4 (0.7)	0 (0.0)		
Condition on study entry										
Est. blood loss, median (IQR)	750 (750)	975 (750)	1000 (500)	1200 (500)	1000 (850)	1000 (850)	700 (500)	781 (700)		
Mean arterial pressure < 60	17 (10.8)	32 (16.0)	181 (29.9)	321 (38.5)	188 (40.1)	158 (51.6)	150 (26.1)	42 (28.4)		
Unconscious	2 (1.3)	7 (3.4)	27 (4.5)	41 (5.0)	6 (1.3)	12 (3.9)	1 (0.2)	1 (0.7)	14 (10.1)	10 (8.3)
Outcomes										
Blood loss, median (IQR)	500 (450)	250 (400)	370 (550)	50 (175)	500 (550)	150 (200)	480 (450)	400 (370)		
Mortality	2 (1.3)	0 (0.0)	38 (6.3)	29 (3.5)	13 (2.8)	5 (1.6)	9 (1.6)	5 (3.4)	35 (28.9)	27 (19.4)

^*a*^*Excluding weeks pregnant ≤ 24 weeks. *

^*b*^*Some data not available from Maknikar 2012.*

Four studies reported blood loss measured by a closed-end calibrated plastic drape. In three of these sites, median measured blood loss value was reduced by 50% or more in the NASG intervention group versus the control group. While the Miller 2006, Miller 2010 and Magwali 2012 trials all reported such decline, the median measured blood loss in Miller 2012 represented a smaller decrease across study arms. Maknikar 2012 did not report blood loss.

**Figure 2:**
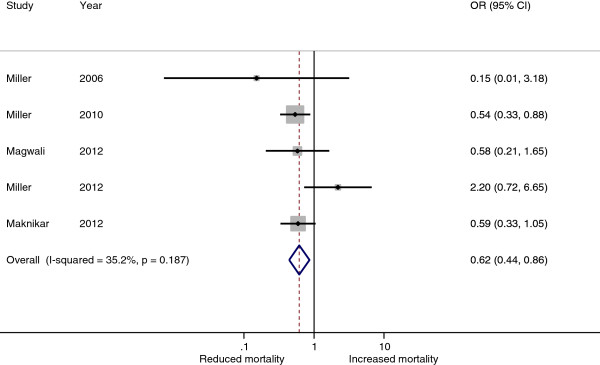
Forest plot describing odds ratios for mortality from hypovolemic shock secondary to obstetric hemorrhage in NASG intervention phase vs. control phase, all study participants (n=3563).

All five studies presented odds ratios for the effect of NASG intervention on mortality (Figure 2). In four of the studies, NASG intervention was associated with a reduced odds of mortality (OR range 0.15 – 0.59), however, this effect reached statistical significance for only one study (Miller 2010) [12]. Results from Miller 2012 were not consistent with this pattern; for this study, NASG intervention was associated with a two-fold increased odds of mortality. However, this finding was not statistically significant (OR 2.20, 95% CI 0.72 – 6.65). Across the five studies significant heterogeneity of effect was not identified (Q 6.16, p = 0.187), and the I^2^ estimate was also not significant (95% CI 0 – 76). Among the 3,563 women included in the five studies, the POR for mortality was 0.62 (95% CI 0.44 – 0.86), suggesting that NASG treatment was associated with a 38% significantly reduced odds of mortality.

### Subanalysis of women with most severe shock

**Figure 3:**
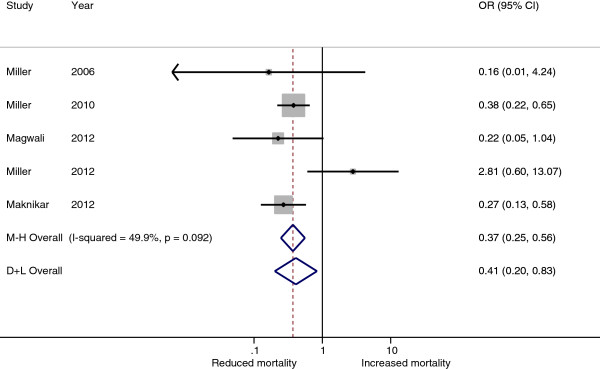
Forest plot describing odds ratios for mortality from hypovolemic shock secondary to obstetric hemorrhage in NASG intervention phase vs. control phase, study participants in most severe shock (n=1229).

Across the studies, 1,229 women were considered to be in the most severe condition, defined as MAP < 60 mmHg or unconscious on study entry (Table 2). Fifty-two percent (51.7%) received the NASG plus standard OH care while 48.3% received standard OH care only. The characteristics of the subsample were largely similar to the overall sample, however estimated blood loss at study entry appeared slightly higher. In three of the four studies that reported blood loss, median measured blood loss was reduced by at least 55% in the NASG intervention phase compared to the control phase. The difference in median measured blood loss between intervention groups in the Miller 2012 study was much smaller. Effect estimates from the studies largely indicate a protective effect of the NASG on mortality, with odds ratios ranging from 0.16 – 0.38 (Figure 3), and two of the studies reached statistical significance (Miller 2010, and Maknikar 2012). Again, the result from Miller 2012 study was inconsistent with the other studies (OR 2.81, 95% CI 0.60 – 13.07). The Q statistic suggested significant heterogeneity of effect across studies (Q 7.95, p = 0.092) using a conservative threshold. The POR utilizing a random effects model was 0.41 (95% CI 0.20 – 0.83), indicating that NASG treatment was associated with a 59% reduced odds of mortality from obstetric hemorrhage among the women with the most profound shock.

**Table 2:** Selected characteristics of study participants for studies included in combined analysis of NASG, participants in most severe shock n = 1,229

	**Miller 2006**		**Miller 2010**		**Magwali 2012**		**Miller 2012**		**Maknikar 2012**^**b**^	
	**Control**	**NASG**	**Control**	**NASG**	**Control**	**NASG**	**Control**	**NASG**	**Control**	**NASG**
	**n = 18**	**n = 35**	**n = 183**	**n = 327**	**n = 190**	**n = 162**	**n = 150**	**n = 42**	**n = 53**	**n = 69**
Demographics										
Age mean (SD)	25.9 (5.3)	26.9 (5.8)	30.1 (6.5)	30.2 (6.3)	28.3 (5.8)	28.0 (6.0)	26.7 (6.9)	26.8 (7.0)		
Parity mean (SD)	2.6 (2.5)	1.5 (1.8)	4.8 (3.1)	4.4 (3.3)	2.4 (1.8)	2.4 (1.9)	2.3 (2.2)	2.3 (2.0)		
Weeks pregnant ^*a*^, mean (SD)	38.4 (3.1)	39.2 (1.2)	37.7 (2.8)	37.1 (3.2)	36.7 (3.7)	37.4 (3.4)	36.3 (2.7)	36.4 (3.0)		
Definitive diagnosis										
Uterine atony	7 (38.9)	12 (34.3)	57 (31.2)	79 (24.2)	28 (14.7)	31 (19.1)	20 (13.3)	6 (14.3)		
Complications of abortion	3 (16.7)	9 (25.7)	12 (6.6)	36 (11.0)	67 (35.3)	50 (30.9)	31 (20.7)	14 (33.3)		
Placenta previa	1 (5.6)	0 (0.0)	11 (6.0)	22 (6.7)	4 (2.1)	6 (3.7)	10 (6.7)	0 (0.0)		
Placental abruption	2 (11.1)	0 (0.0)	19 (10.4)	49 (15.0)	13 (6.8)	13 (8.0)	6 (4.0)	2 (4.8)		
Ectopic pregnancy	0 (0.0)	9 (25.7)	17 (9.3)	26 (8.0)	14 (7.4)	2 (1.2)	4 (2.7)	5 (11.9)		
Molar pregnancy	0 (0.0)	1 (2.9)	1 (0.6)	6 (1.8)	1 (0.5)	0 (0.0)	2 (1.3)	0 (0.0)		
Ruptured uterus	2 (11.1)	1 (2.9)	20 (10.9)	19 (5.8)	14 (7.4)	7 (4.3)	2 (1.3)	2 (4.8)		
Placenta accreta	0 (0.0)	0 (0.0)	4 (2.2)	5 (1.5)	1 (0.5)	1 (0.6)	2 (1.3)	1 (2.4)		
Lacerations/Genital trauma	1 (5.6)	1 (2.9)	4 (2.2)	24 (7.3)	10 (5.3)	6 (3.7)	31 (20.7)	3 (7.1)		
Retained placenta	0 (0.0)	1 (2.9)	38 (20.8)	56 (17.1)	37 (19.5)	44 (27.2)	39 (26.0)	9 (21.4)		
Other	2 (11.1)	1 (2.9)	0 (0.0)	3 (0.9)	1 (0.5)	1 (0.6)	3 (2.0)	0 (0.0)		
Condition on study entry										
Est. blood loss, median (IQR)	1500 (750)	1500 (1150)	1500 (1000)	1500 (800)	1000 (800)	1000 (500)	700 (500)	800 (700)		
Mean arterial pressure < 60	17 (94.4)	30 (93.8)	181 (98.9)	321 (98.2)	188 (99.0)	158 (97.5)	150 (100.0)	42 (100.0)		
Unconscious	2 (11.1)	6 (18.2)	27 (15.1)	41 (12.9)	6 (3.2)	12 (7.4)	1 (0.7)	1 (2.4)	53 (100.0)	69 (100.0)
Outcomes										
Blood loss median (IQR)	555 (700)	250 (350)	360 (500)	55 (175)	600 (520)	150 (200)	480 (480)	430 (370)		
Mortality	1 (5.6)	0 (0.0)	34 (18.6)	26 (8.0)	10 (5.3)	2 (1.2)	4 (2.7)	3 (7.1)	31 (58.5)	19 (27.5)

^*a*^*Excluding weeks pregnant ≤ 24 weeks.*

^*b*^*Some data not available from Maknikar 2012.*

### Sensitivity analyses

The POR calculated through sequentially excluding one trial from the analysis ranged from POR 0.55 (0.39 – 0.78) when Miller 2012 was excluded to POR 0.69 (0.44 – 1.08) when Miller 2010, the largest trial, was excluded (Table 3). Similar results were observed for the subgroup of women with severe shock, where the most protective effect estimate was observed when excluding Miller 2012 (POR 0.32, 95% CI 0.21 – 0.49), ranging through POR 0.51 (0.17 – 1.49) where Maknikar 2012 was excluded.

**Table 3:** Sensitivity analysis: pooled odds ratios for mortality from hypovolemic shock secondary to obstetric hemorrhage in NASG intervention phase versus control phase, sequentially removing one study from pooled analysis, for all study participants (n = 3563) and study participants in most severe shock (n = 1229)

**Excluded study**	**All Participants**		**Most severe shock**	
	**OR**	**95% CI**	**OR**	**95% CI**
Miller 2006	0.63	0.45 – 0.88	0.43	0.20 – 0.94
Miller 2010	0.69	0.44 – 1.08	0.45	0.13 – 1.55
Magwali 2012	0.62	0.44 – 0.88	0.47	0.20 – 1.09
Miller 2012	0.55	0.39 – 0.78	0.32	0.21 – 0.49
Maknikar 2012	0.63	0.42 – 0.95	0.51	0.17 – 1.49
Combined	0.62	0.44 – 0.86	0.41	0.20 – 0.84

## Discussion

Overall the combined results and pooled effect estimates from this combined analysis suggest that NASG intervention at tertiary facilities is associated with a reduced odds of death for women with hypovolemic shock secondary to obstetric hemorrhage. For all women we observed a statistically significant 38% reduction in the odds of death, and for women in the most severe shock we observed a 59% protective effect. The studies included in the review all had similar design, intervention, and eligibility criteria. Small differences were observed in the distribution of hemorrhage etiology.

Across the included studies, the outcomes of measured blood loss and mortality were different for Miller 2012 compared to the other four trials. The authors had some reservations about including this trial into the analysis due to quality concerns expressed by the research team. During the pre-intervention phase there was over-enrollment of mild cases. A review of all facility-based OH-related maternal deaths revealed that the proportion of actual maternal deaths enrolled in the study was low and varied by intervention phase. During the control phase, 37.5% of actual OH-related maternal deaths were captured by the study, whereas in the intervention phase, 60.5% of these deaths were captured. It is therefore not surprising that excluding Miller 2012 during the sensitivity analysis revealed the strongest effect for the NASG intervention: POR 0.55 (95% CI 0.39 – 0.78) among the full sample and POR 0.32 (95% CI 0.21 – 0.49) for women in the most severe shock. We chose not to exclude this particular trial because the results from this trial may contribute an important conservative estimate of the NASG under certain conditions of use.

While the results of this analysis suggest a reduction in the odds of mortality associated with NASG intervention, it is important to consider the specific methodological decisions that went into this analysis. Our protocol defined a 10% type error rate as the threshold for considering whether heterogeneity of effect existed across the findings from the component studies. The Q statistics generated from our models indicated that results were not significantly heterogenous for the overall sample (p = 0.187), but that they were for the subsample of women in the most severe shock (p = 0.092). Thus, the analysis method differed across the samples; we utilized a fixed effect method for the overall sample and a random effect method for the subsample. The threshold that we utilized to determine significant heterogeneity was conservative, however, it could be argued that a more conservative approach was warranted for this analysis due to the small number of individual studies trialing the NASG and the increased likelihood of committing a type II error for the Q and I^2^ statistics [26,27]. Such an approach could have included pursuing a random-effects analysis of the overall sample; we explored the random-effects analysis and while the findings from this analysis were similar in their clinical significance, the wider confidence interval was not statistically significant (POR 0.66, 95% CI 0.41 – 1.07). However, interpretation of these results should take into consideration the assumptions inherent to each of these methods and the quality concerns around the one study that is responsible for the observed heterogeneity of effect. Furthermore, sensitivity analyses demonstrated the pooled effect estimates and their confidence intervals for both fixed and random methods to be equal and support a statistically significant reduction in odds of mortality when excluding this study from the analysis (not shown).

Strengths of this combined analysis include the comprehensive inclusion of NASG tertiary facility trials; because the research team is the only known group to have conducted any evaluations of the NASG to date, and the only known group to provide technical assistance and training to groups adopting the NASG into their obstetric hemorrhage protocols, we feel relatively confident that there are no trials missing from this analysis. Furthermore, inclusion and exclusion criteria are consistent across studies, and all studies utilized the same comparison groups of NASG plus standard care for OH versus standard care for OH only. Finally, we did not limit the included trials to those that have been published, allowing for inclusion of more data; three trials whose results were peer-reviewed for presentation at conferences were included.

The primary limitation of this analysis is that the data do not come from randomized controlled trials, but observational studies. Furthermore, there are very few trials that have been conducted utilizing the NASG, and the trials that have been conducted are small and contain few deaths. Because all trials have been implemented by the same group (UCSF) or in collaboration with this group, it is possible that any small but systematic biases present across these studies may have become exaggerated within our pooled effect estimates. Additionally, three of the five studies included in the combined analysis were not peer-reviewed full manuscripts; they were presented solely at a conference. There were minimal differences in populations across trials in terms of participant’s characteristics at baseline or the variations in hemorrhage etiologies.

## Conclusions

While these results support implementation of the NASG in tertiary care facilities where women continue to suffer from delays in receiving definitive treatment, it is likely that the NASG may decrease mortality if applied at the community or primary health care level before transfer to the referral/tertiary-facility. The introductory phases of the Magwali 2012 and Miller 2012 trials included in this analysis were implemented in preparation for a cluster randomized controlled trial (CRCT), a more robust methodology, set in primary health care centers conducted from June 2007 through May 2012. In conjunction with the results presented in this analysis, the findings from the CRCT and any subsequent robust studies will help researchers and clinicians to better understand the utility of the NASG in preventing mortality from hypovolemic shock secondary to obstetric hemorrhage at different levels of the health care system.

## Abbreviations

BPM: Beats per minute; CEmOC: Comprehensive emergency obstetric care; MAP: Mean arterial pressure; NASG: Non-pneumatic anti-shock garment; OH: Obstetric-hemorrhage; PPH: Postpartum hemorrhage; POR: Pooled odds ratio; SBP: Systolic blood pressure; UCSF: University of California, San Francisco.

## Competing interests

The authors declare that they have no competing interests.

## Authors’ contributions

AE conducted literature review, data analysis, table preparation and drafted the manuscript. EB participated in the design of the analysis, conducted literature review and reviewed the manuscript. JG reviewed the manuscript. SM participated in the design of the analysis, and reviewed the manuscript. All authors read and approved the final manuscript.

## Pre-publication history

The pre-publication history for this paper can be accessed here:

http://www.biomedcentral.com/1471-2393/13/208/prepub
